# Dihydrochalcone Derivatives from *Populus balsamifera* L. Buds for the Treatment of Psoriasis

**DOI:** 10.3390/ijms21010256

**Published:** 2019-12-30

**Authors:** Audrey Bélanger, Alexe Grenier, François Simard, Isabelle Gendreau, André Pichette, Jean Legault, Roxane Pouliot

**Affiliations:** 1 Centre de Recherche en Organogénèse Expérimentale de l’Université Laval/LOEX, Axe Médecine Régénératrice, Centre de Recherche du CHU de Québec—Université Laval, Québec, QC GIJ 1Z4, Canada; audrey1_belanger@uqac.ca (A.B.); alexe.grenier.1@ulaval.ca (A.G.); isabelle.gendreau.1@ulaval.ca (I.G.); 2 Faculté de Pharmacie, Université Laval, Québec, QC G1V 0A6, Canada; 3 Laboratoire d’Analyse et de Séparation des Essences Végétales (LASEVE), Département des Sciences Fondamentales, Université du Québec à Chicoutimi, Chicoutimi, QC G7H 2B1, Canada; Francois1_Simard@uqac.ca (F.S.); Andre_Pichette@uqac.ca (A.P.); Jean_Legault@uqac.ca (J.L.)

**Keywords:** natural products, dihydrochalcone, balsacone, tissue engineering, skin substitute, psoriasis, in vitro study

## Abstract

Psoriasis is a skin disorder characterized by epidermal hyperplasia, hyperkeratosis, and inflammation. The treatments currently available on the market only improve patients’ quality of life and are associated with undesirable side effects. Thus, research leading to the development of new, effective, and safer therapeutic agents is still relevant. *Populus balsamifera* L. buds were used traditionally by Native Americans to treat various skin pathologies such as eczema and psoriasis. In this study, the antipsoriatic activities of dihydrochalcone derivatives from *Populus balsamifera* L. buds, known as balsacones, were investigated. The experiments were performed in vitro using a psoriatic skin substitute model. Also, anti-inflammatory and antioxidant activities were investigated. The tested balsacones showed promising antipsoriatic properties by slowing down cell growth and by regulating the expression of involucrin, loricrin, and Ki67 better than methotrexate in psoriatic substitutes. All five tested compounds could be an effective topical treatment for psoriasis, with promising anti-inflammatory and antioxidant actions that may contribute to clinical improvement in patients with psoriasis.

## 1. Introduction

The skin barrier function depends on the structure and composition of the uppermost layer of the epidermis, the *stratum corneum* (SC), which plays key roles in immune surveillance, homeostasis, and in preventing the penetration of microbial products and allergens. The process of keratinization is characterized by a series of morphological changes in the keratinocytes. Briefly, there is a loss of adhesion of basal cells to the basement membrane and a progression into spinous cells, which in turn form a granular layer that contains a new organelle, keratohyalin granules. These cells eventually differentiate and form a cornified cell envelope (CE), resulting in the formation of the most superficial layer of the skin, the SC. Covalent bindings between ceramide lipids and proteins in the epidermis are responsible for the orderly arrangement of extracellular lipids in lamellae, which contributes to the protective barrier function of the CE [1,2]. 

Psoriasis is an erythematous-squamous dermatitis affecting 2% to 3% of the world’s population [3]. At a cellular level, psoriasis is characterized by the hyperproliferation (hyperkeratosis) and abnormal differentiation of keratinocytes resulting in the thickening of the epidermis (acanthosis) and the absence of the granular layer (agranulose) [4,5,6]. The greatly increased keratinocyte proliferation causes scaling on the skin’s surface, which is often covered with loose, silver-colored scales that may be itchy and painful [7]. The pathogenesis of psoriasis also involves the infiltration of immune cells into the dermis and epidermis, causing the secretion of inflammatory mediators [8]. Cytokines secreted by immune cells stimulate the keratinocytes, which in turn triggers the formation of lesion plaques [9,10].

At a biochemical level, the accelerated growth of skin cells will generate problems in cell differentiation, which consequently alters the expression of several proteins involved in the formation of the CE and thereby affects the skin barrier function by increasing the permeability of the skin [11]. Although the complete identification of the specific mechanisms controlling epidermal stratification and homeostasis is still unfolding, some of the proteins crucial for these processes have been identified. Indeed, loricrin (LOR) and involucrin (IVL) are major protein markers of cell differentiation that have an important role in the epidermal barrier. LOR comprises more than 70% of the CE, reinforces the CE and enhances its defensive barrier function [12]. The interaction of LOR with keratin intermediate filaments provides flexibility to the CE. The expression of this late differentiation markers depends on the keratohyalin granules, which are present in a lesser amount in psoriatic skin than in normal skin due to abnormal keratinocyte differentiation [13,14]. IVL, an early component in the assembly of the CE, is synthesized in the *stratum spinosum* and provides a scaffold for the CE [12,15,16]. Because the synthesis of IVL is correlated with cell migration beyond the basal layer, the amount of IVL is markedly increased in inflammatory skin diseases such as psoriasis [17,18].

**Figure 1:**
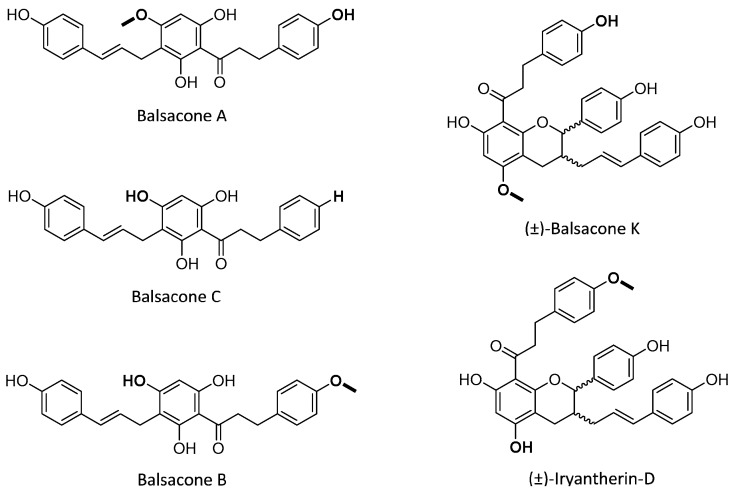
Structure of balsacone A, balsacone B, balsacone C, (±)-balsacone K, and (±)-iryantherin-D.

Although there are many treatments that improve the quality of life of patients, the main problems with these treatments are the side effects [4,19,20]. Thus, the identification of compounds possessing antipsoriatic activities with few adverse effects still remains of great interest in the field of dermatology. Natural products are known to possess a wide range of beneficial effects on human health and the discovery of many new drugs results from the identification of bioactive natural products [21,22]. The potential of flavonoids to treat psoriasis in relation to their anti-inflammatory activity was recently reviewed [23]. In this work, the potential of dihydrochalcone derivatives recently isolated from *Populus balsamifera* L. buds [24,25,26] to treat psoriasis was evaluated. Indeed, Canadian Aboriginals used *Populus balsamifera* L. buds in their traditional medicine for the treatment of skin diseases like eczema and psoriasis [27,28]. In vitro screening studies of this unique series of dihydrochalcone derivatives revealed antibacterial activity against both *Staphylococcus aureus* and clinical isolates of methicillin-resistant *Staphylococcus aureus* (MRSA) [24,26,29,30]. However, little work has been conducted on the elucidation of their biological and pharmacological potentials. Nowadays, tissue engineering makes it possible to study pathologies such as psoriasis more effectively by using psoriatic skin cells to reconstruct skin substitutes that mimic the phenotypic characteristics of dermatitis. In this study, a representative psoriatic skin model based on a self-assembly approach, which shows in vivo features of psoriasis, has been used to examine the expression of proteins involved in the formation of the CE [31,32,33,34]. Furthermore, the anti-inflammatory and antioxidant activities of various compounds were evaluated. Methotrexate (MTX), was used in this study as a reference compound. MTX is a systemic drug commonly used to treat moderate to severe psoriasis [35]. Balsacone A (BA), balsacone B (BB), balsacone C (BC), (±)-balsacone K (BK), and (±)-iryantherin-D (BD) (Figure 1) exhibited potent in vitro antipsoriatic activities.

## 2. Results

### 2.1. Antiproliferative Potential

The antiproliferative potential of these compounds was evaluated using a sulforhodamine B (SRB) assay. The results presented in Table 1 are expressed as the concentration inhibiting fifty percent of cell growth (IC_50_). Dihydrochalcone derivatives inhibited keratinocyte cells growth with IC_50_ ranging from 23 to 128 µM. These concentrations were used in further experiments to treat healthy (H) and psoriatic (P) skin substitutes.

### 2.2. Macroscopic and Histological Analyses

**Figure 2:**
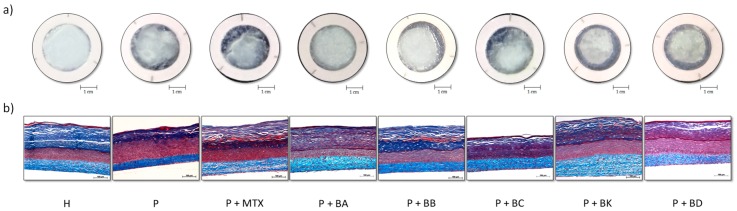
Macroscopic (**a**) and histological (**b**) analyses of tissue-engineered skin substitutes. Photos are of healthy (H) and psoriatic (P) substitutes and psoriatic substitutes treated with methotrexate (MTX), balsacone A (BA), balsacone B (BB), balsacone C (BC), (±)-balsacone K (BK) or (±)-iryantherin-D (BD).

Macroscopic and histological analyses with Masson’s trichrome staining demonstrate the differences between H and P substitutes reconstructed in vitro (Figure 2). Macroscopic analyses confirmed the integrity of skin substitutes reconstructed according to the self-assembly method (Figure 2a). Macroscopic photos of H substitutes allow us to observe the epidermis which is opaque, smooth, and uniform, covering the entire seeding area, and thus, demonstrating the normal proliferation and differentiation of keratinocytes. Macroscopic photos of P substitutes show excessive proliferation and the early differentiation of keratinocytes since some areas of the epidermis are whitish, meaning there is a greater number of cornified cells. Histological analyses showed that P substitutes have a thicker living epidermis than H substitutes (Figure 2b). P substitutes treated with one of the five compounds (BA, BB, BC, BK or BD) or MTX, used as a control treatment for comparison, seem to have a living epidermis thickness comparable to the H substitutes.

### 2.3. Living Epidermis Thickness

**Figure 3:**
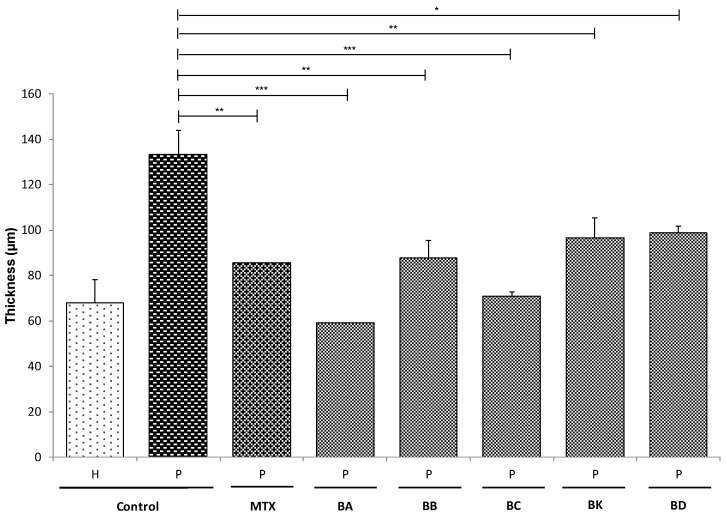
Epidermal thickness. Measurements of the living epidermis thickness of healthy (H) and psoriatic (P) skin substitutes without treatment (control) or treated with methotrexate (MTX), balsacone A (BA), balsacone B (BB), balsacone C (BC), (±)-balsacone K (BK), or (±)-iryantherin-D (BD). Statistical significance was determined using a one-way ANOVA (*p* < 0.05). Data presented are means ± SD; *N* = 2, *n* = 2, 20 measurements per condition for each condition, except balsacone A, where *N* = 1, *n* = 2. (*** *p* < 0.001, ** *p* < 0.01, * *p* < 0.05).

The effects of the different treatments on epidermal morphogenesis were studied by comparing the thickness of the different tissues at day 21 of the air–liquid interface (A/L), because tissue culture during 21 days at A/L promotes and ensures a full differentiation and stratification of the epidermis. Living epidermis thickness was measured from the histological analyses (Figure 2b). The thickness of psoriatic living epidermis was found to be significantly thicker compared with the healthy living epidermis (P: 133 ± 10 μm, H: 68 ± 10 μm, *p* < 0.001) (Figure 3). The epidermis thickens due to keratinocyte hyperproliferation. However, when treated with either the standard treatment MTX or BA, BB, BC, BK or BD, there was a significant reduction in the thickness of the living epidermis of P substitutes (P + MTX: 85.53 ± 0.04 μm, P + BA: 59.02 ± 0.06 μm, P + BB: 88 ± 8 μm, P + BC: 71 ± 2 μm, P + BK: 96 ± 9 μm, P + BD: 99 ± 3 μm vs. P: 133 ± 10 μm) (Figure 3), and a better keratinocyte differentiation compared with the untreated P substitutes, as seen in Figure 2b. For the H substitutes, no significant differences were observed between the untreated and the treated skin models (Supplementary Figure S1).

### 2.4. Immunofluorescence Staining

**Figure 4:**
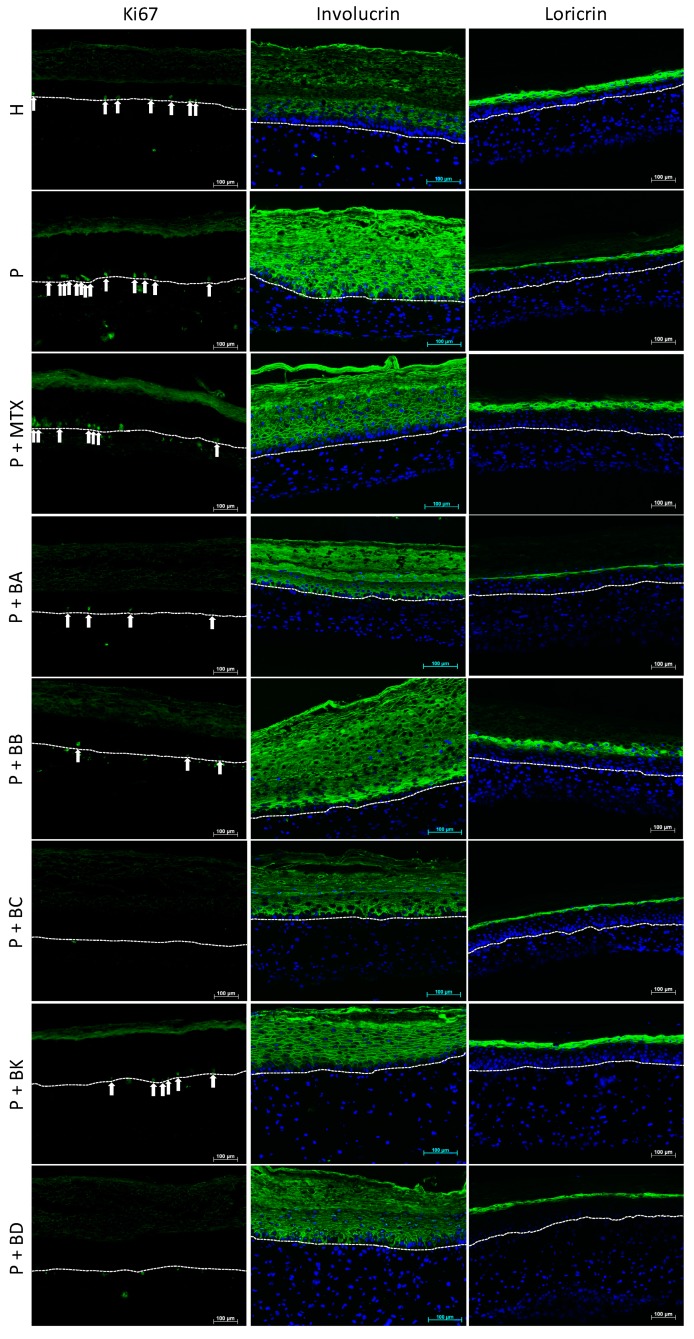
Immunofluorescence staining. Expression of Ki67 (green, stained cells are indicated with arrows), involucrin (green) and loricrin (green) monitored in healthy (H) or psoriatic (P) skin substitutes treated or not with methotrexate (MTX), balsacone A (BA), balsacone B (BB), balsacone C (BC), (±)-balsacone K (BK), and (±)-iryantherin-D (BD). Nuclei were stained with Hoechst (blue). Three substitutes of each condition were analyzed and the results were confirmed with two independent experiments. (Scale bar = 100 μm).

Immunofluorescence stainings were performed for proliferation (Ki67) and differentiation (IVL, LOR) markers (Figure 4). Ki67 staining showed hyperproliferation in the P substitutes, as compared with the control (H substitutes). In Figure 4, thirteen proliferative basal cells were stained in the P substitute compared to seven in the H substitute. When treated with MTX, P substitutes showed a number of proliferative cells that was more similar to the H substitutes. The five balsacones also helped to restore the proliferative profile in P substitutes, since only a few basal cells were positive for Ki67.

The stainings of IVL, an early differentiation marker, and LOR, a late differentiation marker, were performed to evaluate the differentiation process. As can be observed in Figure 4, the early differentiation marker (IVL) is overexpressed in P substitutes (without treatment), while the late differentiation marker (LOR) is underexpressed, compared with H substitutes. When treated either with the standard treatment MTX or with BA, BB, BC, BK or BD, the expression of IVL and LOR seems partially or fully restored, suggesting a normalization of the differentiation process. The fluorescence intensity analyses of microscopy images have confirmed an increase in pixel intensity for LOR of over 100% when P substitutes were treated with BB or BK, compared with untreated P substitutes, while MTX treatment increased the presence of LOR up to 66% compared with the control. The pixel intensity of IVL, when substitutes were treated with MTX, decreased to 41% compared with untreated P substitutes, while BA and BC treatments led to a fully restored IVL expression as found in a healthy phenotype. Treatments with BK, BB or BD revealed a partial expression of IVL (respectively an 89%, 40%, and 12% decrease in pixel intensity compared with P substitutes), suggesting that P substitutes treated with one of BA, BB, BC, BK or BD are closer to a healthy phenotype. According to these analyses, BA, BB, BC, and BK seem to act even better than MTX on the differentiation process.

### 2.5. Anti-Inflammatory and Antioxidant Properties

Anti-inflammatory activity, a characteristic of interest for the treatment of psoriasis, was investigated by measuring nitric oxide (NO) production induced by lipopolysaccharide (LPS) on macrophage RAW 264.7 cells. The most potent compounds were BK and BB with IC_50_ of 10.49 ± 0.07 µM and 13.3 ± 0.8 µM respectively (Table 2). In order to assess whether the decrease in NO could be attributed to an increase in cell death or to NO production inhibition, cell viability was measured as a function of the balsacone concentration. Only high concentrations (40 μM) of BB and BK caused a statistically significant reduction in cell viability (data not shown).

Balsacones demonstrated antioxidant activity by significantly inhibiting the oxidation of DCFH induced by tert-butyl hydroperoxide (*t*-BuOOH) in WS1 cells. Most potent compounds were BB and BC with an IC_50_ of 1.05 ± 0.09 µM and 1.10 ± 0.08 µM respectively (Table 2).

## 3. Discussion

### 3.1. Histological Analysis: The New Compounds Regulated the Differentiation of Psoriatic Keratinocytes

Acanthosis is a thickening of the skin, more specifically an increase in the thickness of the spinous layer of the living epidermis due to the exaggerated multiplication of keratinocytes found in the basal layer [4]. Histological analyses show that P substitutes presented thickening of the living epidermis (Figure 2b), which is in accordance with the acanthotic characteristics of the pathology. Masson’s trichrome staining revealed that P substitutes displayed hyperproliferation and disorganization of the keratinocytes, in contrast to the H substitutes. 

The five compounds studied showed a strong potential for normalizing psoriatic keratinocyte proliferation and for improving their differentiation even going so far as to be comparable to the positive control MTX. In fact, Figure 3 presents the thickness of the living epidermis treated with one or another of the balsacones and all of them presented results similar to the use of MTX. BA and BC effected an improvement of 56% and 47% respectively compared to 36% for MTX; these are very interesting results for potential antipsoriatic candidates [36]. Interestingly, these five compounds have a similar efficacy to MTX on the proliferation of psoriatic keratinocytes at doses up to 15, 9, 5, 16, and 31 times lower with BA, BB, BC, BK, an BD, respectively.

### 3.2. Improvement of the Epithelial Phenotype

#### 3.2.1. Prevention of Hyperproliferation of Psoriatic Keratinocytes

To further investigate the biological role of balsacone family in psoriasis, the effect of balsacones on keratinocytes proliferation markers, such as Ki67, was examined. Ki67 is a proliferation marker that appears in the basal layer of the epidermis and it is known to be overexpressed in the psoriatic epidermis [37]. In this study, P substitutes showed a higher number of cells stained by Ki67 compared with H substitutes (Figure 4). However, when comparing P substitutes before and after they received treatment, P substitutes treated with one or another of the five compounds or with MTX showed a reduction in Ki67 expression and showed similarities to the H substitutes. These analyses suggest that the compounds influence the psoriatic keratinocyte cell cycle and that their efficacy is comparable to MTX for the proliferative process. Indeed, the compounds prevented the hyperproliferation observed in psoriasis.

#### 3.2.2. Improvement of Barrier Protein Expression

To investigate more deeply whether treatments did indeed improve these psoriatic cells’ differentiation and proliferation, differentiation markers were examined. Immunostaining was performed to determine the effect of compounds from *Populus balsamifera* L. buds on the IVL expression of keratinocytes. The altered differentiation of psoriatic keratinocytes is characterized by an upregulation of early differentiation markers, like IVL [18]. Results indeed showed an upregulation of IVL in P substitutes when compared with H substitutes (Figure 4). However, the five balsacones may play a positive role in regulating epidermal differentiation, as observed in treated P substitutes compared with untreated P substitutes. These results are similar to those obtained with P substitutes treated with MTX, demonstrating that the balsacones appear to be as effective on cell differentiation as this standard drug for the treatment of psoriasis. 

The expression of LOR, a late differentiation marker, was also analyzed since its expression is downregulated in the pathology [13]. In this study, we found the marker to be underexpressed in the living epidermis of the P substitutes as compared with H substitutes (Figure 4), which is in accordance with in vivo observations. Immunostaining confirmed an increased expression of LOR in P substitutes after treatment with one or another of the five balsacones, compared with untreated P substitutes. P substitutes treated with MTX also showed an increase in expression of the marker compared with controls. These dihydrochalcone derivatives have an antipsoriatic potential since they induce an increase in LOR synthesis, on which the formation of the granular layer depends (quantity of keratohyalin granules), and which is usually greatly reduced in, or even absent from, the psoriatic epidermis [4]. Our results demonstrate that the protein expression of keratinocytes is regulated by BA, BB, BC, BK, and BD, where BA and BC seem to affect the expression of an early differentiation marker (IVL), in contrast to BB and BK, which appear to have an impact on the expression of a late differentiation marker (LOR). According to these results, the compounds belonging to the balsacone family could improve keratinocyte differentiation.

### 3.3. Diminution of Macrophage-Induced Inflammation and Potential Antioxidant Effect

In addition to improving the epithelial phenotype by normalizing the epithelial differentiation process, all tested compounds have demonstrated its potential for decreasing macrophage-induced inflammation. NO is a key mediator of the inflammatory response. After exposure to bacterial LPS, macrophages respond with the release of NO, which can trigger a number of pathophysiological consequences including tissue damage. Hence, measuring the inhibition of NO production in LPS-stimulated cells is a widely used experimental method for examining the anti-inflammatory effects of compounds. All balsacones tested have shown an interesting anti-inflammatory potential. BK was the most potent, as it inhibited NO production with an IC_50_ value of 10.49 ± 0.07 μM, followed by BB with an IC_50_ of 13.3 ± 0.8 µM.

NO production has also been investigated in other studies on antipsoriatic agents, since the inducible nitric oxide synthase (iNOS) is overexpressed in psoriatic skin and this could suggest an upregulation in NO synthesis [38,39]. NO is produced when L-arginine is converted into L-citrulline by NO synthases (NOS) including iNOS. A study on the antipsoriatic potential of a natural product containing polyphenols such as trans-resveratrol, a polyphenolic extract from *Picea mariana* bark, showed that 250 and 500 μg/mL of the extract could decrease the NO production induced in normal and psoriatic keratinocytes by TNF-α [40]. A decrease in NO production was also reported in a study on MTX therapy, the control treatment in the present study [41]. The ability to reduce NO production seems to be an important characteristic for an antipsoriatic treatment since this suggests a potential anti-inflammatory action. As an inflammatory skin disease involving immune cells such as macrophages and T-cells, psoriasis could benefit from such a quality [42,43]. The results obtained regarding the anti-inflammatory potential of the five balsacones suggest the efficacy of these compounds as a potential treatment.

Several studies have suggested the involvement of oxidative stress in psoriasis [44,45,46]. Oxidative stress, often due to an imbalance between reactive oxygen species (ROS) and antioxidants in the body, can eventually lead to molecular damage. ROS are usually second messengers in the ligand/receptor-initiated pathways, which can lead to the production of inflammatory mediators, cytokines and growth factors [47]. Thereby, this ROS imbalance can play a role in inflammatory pathologies like psoriasis. Several studies have also found increased levels of oxidative stress markers, confirming the ROS imbalance in the pathology [48,49,50,51]. ROS can also regulate cellular processes such as proliferation, which could have a role in the hyperproliferative characteristic of psoriasis [47]. Therefore, the antioxidant activity of these compounds has an interesting potential for treatments. BB and BC were the more promising compounds for this feature with an IC_50_ of 1.05 ± 0.09 µM and 1.10 ± 0.08 µM respectively. Anti-inflammatory and antioxidant activities make these compounds promising future topical drugs for the treatment of dermatitis, including psoriasis.

In conclusion, psoriasis is a chronic incurable disease, hence the importance of finding effective psoriasis treatments for long-term care and inhibition of the disease manifestations. The 3D psoriatic skin model used in this study features in vivo physiopathological characteristics, and thus accelerates the discovery of promising compounds since this model can serve as a reliable tool in the initial stages of the development of a new, effective and safer antipsoriatic product. This study showed that five plant products from Canada’s boreal forest, which were isolated from *Populus balsamifera* L. buds, namely the compounds BA, BB, BC, BK, and BD, manifested antiproliferative activity in psoriatic keratinocytes by regulating Ki67 antigen expression. These plant-derived polyphenols were also found to regulate human psoriatic keratinocyte differentiation by suppressing the expression of IVL associated with reduced cell proliferation without cell death. Furthermore, these five compounds showed the potential for restoring keratinocyte differentiation by regulating the expression of the LOR marker, as in H skin substitutes. These bioactive compounds have antipsoriatic properties that are as effective as MTX. However, a minimal amount of these plant products is needed compared with MTX, suggesting that the side effects related to their future use on the skin would be negligible. Finally, some compounds isolated from *Populus balsamifera* L. buds showed promising anti-inflammatory actions through the inhibition of NO production (induced by RAW 264.7 cells stimulated with LPS), and presented antioxidant activities.

In light of these results, BB and BC seem to be the balsacones with the greater antipsoriatic effects. BB can regulate Ki67 and LOR expression and has promising anti-inflammatory and antioxidant activities. As for BC, this balsacone can improve the proliferation and differentiation processes as seen with the decrease in epidermal thickness and the regulation of Ki67 and IVL expressions. Moreover, BC showed an antioxidant potential.

Our study supports the use of *Populus balsamifera* L. by the Native Americans of the boreal forest of Canada to treat various diseases and demonstrated, for the first time, that protein expression in keratinocytes is regulated by balsacones. Therefore, the discovery of these natural compounds seems to be promising for the pharmaceutical field. Indeed, in addition to their potential antipsoriatic activities on the differentiation of epidermal cells, their antibacterial properties would protect patients with an altered skin barrier function against pathogens, and prevent both infections and the development of lesions, thus maximally protecting the patient’s skin barrier.

Our results suggest that tested balsacones may be useful in the development of therapeutic agents against psoriasis. Further studies are needed, however, to discover the mechanism of action of these compounds. Additional investigation of the balsacones’ anti-inflammatory mechanisms could be conducted for instance with a 3D organotypic culture model presenting activated T-cell infiltration, based on the self-assembly method as recently developed by Lorthois et al. [52]. Moreover, a study of the *Populus balsamifera* L. buds extract itself, that is to say involving all of the five balsacones at once, could be an interesting avenue of research since all these balsacones seem to have promising, but sometimes different, antipsoriatic effects according to the characteristics studied.

## 4. Materials and Methods

### 4.1. Donors

P skin substitutes were produced with cells from lesional psoriatic biopsies of donors in various stages of plaque-type psoriasis. Psoriatic donors were Caucasian males and females aged 49 and 64 years (♂49 and ♀64). As for the H skin substitutes, biopsies were obtained from healthy donors during breast reduction surgeries. Healthy donors were all Caucasian females aged between 18 and 46 years (♀18, ♀38, and ♀46). Fibroblasts were extracted with the isolation method using thermolysin and collagenase, while keratinocytes were extracted with the isolation method using thermolysin and trypsin [53,54]. Extracted cells were frozen in liquid nitrogen until needed for the following experiments.

### 4.2. Ethical Considerations

All procedures involving donors were in agreement with the Declaration of Helsinki, and performed under the guidelines of the Research Ethics Committee of the “Centre Hospitalier Universitaire (CHU) de Québec” (ethic code: DR-002-1121, protocol renewal approved on 30 January 2019). The donors were given adequate information to allow them to provide written consent.

### 4.3. Compound Preparation

The compounds of *Populus balsamifera* L. buds, BA, BB, BC, BK, and BD, were isolated by the LASEVE as previously described [24,26]. Quantitative ^1^H nuclear magnetic resonance (NMR) spectroscopy was performed to determine the structure of isolated balsacones and to confirm their purity. For all isolated balsacones, the purity obtained was over 95% in order to ensure that the observed effect is accurate and not related to highly active impurities present in the tested samples. The structures of these compounds, isolated as racemates, are given in Figure 1.

Due to the insolubility of the compounds in cell culture media, stock solutions were prepared in DMSO and stored at −20 °C until needed. For the treatments, stock solutions in DMSO were then diluted in cell culture media. The final concentration of DMSO in the culture medium was maintained at 0.1% (V/V) to avoid solvent cytotoxicity. Methotrexate (Injectable USP Methotrexate, 25 mg/mL, Hospira, Montréal, Québec, Canada), commonly used to treat psoriasis, was used as a reference compound. 

### 4.4. Sulforhodamine B Assay

The antiproliferative potential and IC_50_ of the compounds were determined using an SRB (Sigma, Oakville, Ontario, Canada) assay with some minor modifications [55]. Firstly, healthy and psoriatic keratinocytes were seeded in a 96-well plate at 5 × 10^3^ cells/well on a feeder layer of irradiated 3T3 mouse fibroblasts. At day 2, the keratinocytes were treated with increasing concentrations of the compounds, in triplicate. At day 4, cells were fixed with a 50% solution of trichloroacetic acid at 4 °C during two hours. Keratinocytes were then dyed with a 0.1% SRB solution. The IC_50_ was used for further experiments on skin substitutes. 

### 4.5. Tissue-Engineered Skin Production

**Figure 5:**
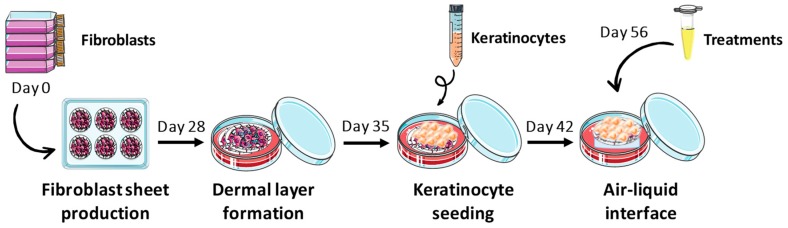
Schematic representation of the self-assembly method for skin substitute production.

In vitro reconstructed H and P skin substitutes were produced according to the partially modified self-assembly method (Figure 5) [31,34]. Briefly, human dermal fibroblasts at their sixth passage were seeded (8 × 10^4^ cells/well) in 6-well cell culture plates, and cultured in the Dulbecco-Vogt modification of Eagle’s medium (DMEM) supplemented with 10% fetal calf serum (FCS; Invitrogen, Burlington, Ontario, Canada), 50 μg/mL ascorbic acid (Sigma, Mississauga, Ontario, Canada), 100 UI/mL penicillin G (Sigma, Oakville, Ontario, Canada) and 25 μg/mL gentamicin (Schering, Pointe-Claire, Québec, Canada) until they formed manipulatable sheets. Then, these sheets were superimposed and incubated to form a new dermal layer. After this period, in order to form an epidermal layer, keratinocytes at their third passage were seeded (1 × 10^6^ cells/well) on the dermal layer and substitutes were cultured in submerged conditions to promote keratinocyte proliferation. Keratinocytes were cultured in a combination of DMEM and Ham’s F12 (3:1), supplemented with 5% Fetal Clone II serum (Hyclone, Scarborough, Ontario, Canada), 50 μg/mL ascorbic acid (Sigma), 5 μg/mL insulin (Sigma), 0.4 μg/mL hydrocortisone (Calbiochem, EMD Biosciences, Gibbstown, NJ, USA), 10^−10^ M cholera toxin (MP Biomedicals, Montréal, Québec, Canada), 10 ng/mL human epidermal growth factor (EGF; Austral Biological, San Ramon, CA, USA), 100 UI/mL penicillin G (Sigma) and 25 μg/mL gentamicin (Schering). Seven days later, skin substitutes were raised to the A/L, and further cultured for 21 days with culture medium exempt of EGF to obtain a stratified epithelium representative of in vivo skin. To treat the H and P skin substitutes, dilutions of the stock solutions were prepared in culture medium at day 14 of the A/L. These dilutions were added directly to the petri dish with the skin substitutes. H and P skin substitutes were treated every two days throughout the week with the diluted compounds. To administer a typical starting dose of MTX for treating severe psoriasis (10 to 25 mg per week), 6.67 mg of solution for injection was added directly to the fresh cultured medium (734 μM), every two days throughout the week. Then, samples of each skin substitute condition at day 21 of A/L were taken, and they were analyzed by histology and immunofluorescence staining. Both cell types were maintained at 37 °C in an incubator containing 8% carbon dioxide (CO_2_) and culture medium was refreshed three times a week. Experiments were performed in triplicate in three independent assays (with fibroblasts and keratinocytes from two or three different donors).

### 4.6. Histology and Immunofluorescence Staining

For histological analyses, samples of each skin substitute were fixed in Histochoice^®^ solution (Amresco, Solon, OH, USA) and embedded in paraffin wax. Five-micrometer-thick sections were stained with Masson’s trichrome using Weigert’s hematoxylin, fuchsin-ponceau, and aniline blue dyes. The thickness of the living part of the epidermis was measured with ImageJ software. Each condition was analyzed in duplicate for two different cell populations and 10 measurements of the live epidermis were taken for each replica (*N* = 2, *n* = 2, 20 measurements for each condition), except for BA, where only one cell population was analyzed (*N* = 1, *n* = 2, 20 measurements).

For immunofluorescence analyses, samples were embedded in Tissue-Tek OCT compound (Somagen Diagnostics, Edmonton, AB, Canada) and frozen in liquid nitrogen. Indirect immunofluorescence assays were performed on 5 µm-thick cryosections permeabilized with acetone. The antibodies used were: rabbit polyclonal anti-Ki67 (IgG) (dilution 1:400, Abcam, Cambridge, UK), mouse anti-involucrin (IgG1) (dilution 1:800; Sigma) and rabbit polyclonal anti-loricrin (IgG) (dilution 1:500, Cedarlane Covance). The secondary antibody was either donkey anti-rabbit IgG (H + L) Alexa 488 (dilution 1:1000, Molecular Probes, Eugene, OR, USA) or goat anti-mouse IgG (H + L) Alexa 488 (dilution 1:1000, Life Technologies, Carlsbad, CA, USA). Cell nuclei were counterstained with Hoechst reagent 33258 (dilution 1:100; Sigma-Aldrich, Saint-Louis, MO, USA) added to the secondary antibody solution. Negative controls consisted of the omission of primary antibodies during the labelling reaction, and frozen sections of normal human skin were used as positive controls.

Histological and immunofluorescence sections were observed under a Zeiss Axio Imager (Carl Zeiss Canada Ltd., Toronto, ON, Canada) with fixed exposure times and enhancement parameters and photographed with an AxioCam ICc1 digital camera. The fluorescence intensity of IVL and LOR staining was measured by pixel intensity using ImageJ software. Briefly, the entire image was analyzed by adjusting the threshold brightness to the same value for each protein. The obtained data were used to compare the fluorescence intensity between H and P substitutes (treated or not).

### 4.7. Measurement of Anti-Inflammatory Activity by Nitrite Quantification

The inhibition of nitric oxide (NO) production by compounds from *Populus balsamifera* L. buds was evaluated as described by Legault et al. [56]. Briefly, the murine macrophages RAW 264.7 (ATCC^®^ TIB-71) were seeded into the wells of 96-well plates (7.5 × 10^4^ cells/well) and cultured in Dulbecco’s modification of Eagle’s medium (DMEM; Multicell Wisent Inc., Saint-Jean-Baptiste, QC, Canada) supplemented with 10% fetal calf serum (Hyclone Laboratories, Logan, UT, USA), 3.7g/L sodium bicarbonate (Multicell Wisent Inc.), MEM vitamin solution (1:100; Corning Cellgro^®^, Manassas, VA, USA), 100 IU/mL penicillin and 100 µg/mL streptomycin (1:100; Corning Cellgro^®^, Manassas, VA, USA). Cells were grown in a humidified atmosphere at 37 °C in 5% CO_2_ for 16 h. Afterwards, cells were treated with the five balsacones at different concentrations and then stimulated with 100 ng/mL LPS. After 24 h, cell-free supernatants were collected and the NO concentration was immediately determined using the Griess reaction. The absorbance was read at 540 nm using an automated Varioskan Ascent plate reader (Labsystems, Milford, MA, USA) and the presence of nitrite was quantified by comparing with a NaNO_2_ standard curve. A maximum concentration of 40 μM was used for the five balsacones and a minimum of eight concentrations (made by serial dilutions) were tested. N-ω-nitro-arginine methyl ester hydrochloride (L-NAME) was used as a positive control at a concentration of 1 mM. Unactivated cells (exposed to media alone) were used as a negative control and activated cells as a positive control.

### 4.8. Evaluation of Antioxidant Activity Using Cell-Based Assay

Antioxidant activity was evaluated using the 2′, 7′-dichlorofluorescein-diacetate (DCFH-DA) assay as described by Girard-Lalancette et al. [57]. To assess antioxidant activity, human skin WS1 fibroblasts (ATCC^®^ CRL-1502) were incubated for 30 min with 100 μL of 5 μM of DCFH-DA in Hanks’ balanced salt solution (HBSS). Afterwards, the cells were incubated for 1 h with serial dilutions of the five balsacones and the positive standard (Quercetin). Then, for the oxidative stress, 100 µL of 200 µM *t*-BuOOH was added and fluorescence was measured immediately and after 90 min. Measurements were performed on an automated plate reader (Fluoroskan Ascent FL, Labsystems, Milford, MA, USA) using an excitation wavelength of 485 nm and an emission wavelength of 530 nm. Antioxidant activity was expressed as the concentration of extract inhibiting 50% (IC_50_) of DCFH oxidation. Indeed, the more a sample is antioxidant, the more the fluorochrome is protected from oxidation, resulting in a reduced detection of fluorescence. 

### 4.9. Statistical Analysis

The antioxidant and anti-inflammatory analyses were carried out with three replicates for each treatment and data were expressed as the mean values of three different experiments. Each assay on skin substitutes was performed two or three times (with fibroblasts and keratinocytes from two or three different donors), each time in triplicate. Experimental results were expressed as mean ± standard deviation. Epidermal thickness measurements were analyzed with a one-way analysis of variance (ANOVA) followed by a Tuckey’s post hoc test. Differences were considered to be significant at a *p*-value < 0.05. The statistical analyses were carried out with R software (v3.2.0, RStudio v0.98.1103, R-core Team 2015).

## Figures and Tables

**Table 1:** Anti-proliferative property of dihydrochalcone derivatives.

Compounds	IC_50_ (μM)
Balsacone A	48
Balsacone B	76
Balsacone C	128
(±)-Balsacone K	45
(±)-Iryantherin-D	23

**Table 2:** Summary of anti-inflammatory and antioxidant activities of balsacones.

Molecule	NO Inhibition IC_50_ (μM) ^1^	Antioxidant IC_50_ (μM) ^2^
Balsacone A	17 ± 1 µM	3.1 ± 0.5 µM
Balsacone B	13.3 ± 0.8 µM	1.05 ± 0.09 µM
Balsacone C	29 ± 1 µM	1.10 ± 0.08 µM
(±)-Balsacone K	10.49 ± 0.07 µM	1.8 ± 0.3 µM
(±)-Iryantherin-D	26.9 ± 0.5 µM	5.1 ± 0.8 µM

^1^ L-NAME (1 mM), used as an anti-inflammatory positive control, produced an inhibition of NO production of 68%; ^2^ Quercetin, used as an antioxidant positive control, had an IC_50_ of 0.082 ± 0.008 µg/mL. All the experiments were carried out in triplicate and presented results are representative of at least two different experiments.

## Supplementary Materials

Supplementary materials can be found at https://www.mdpi.com/1422-0067/21/1/256/s1. Figure S1: Macroscopic (a) and histological (b) analyses of healthy tissue-engineered skin substitutes treated with methotrexate (MTX), balsacone A (BA), balsacone B (BB), balsacone C (BC), (±)-balsacone K (BK) and (±)-iryantherin-D (BD).
