# Supplementary figures and images for: Dihydrochalcone Derivatives from Populus balsamifera L. Buds for the Treatment of Psoriasis
Source: Int J Mol Sci. 2019 Dec 30;21(1):256. doi: 10.3390/ijms21010256 (PMC6981943; doi:10.3390/ijms21010256)

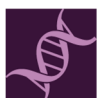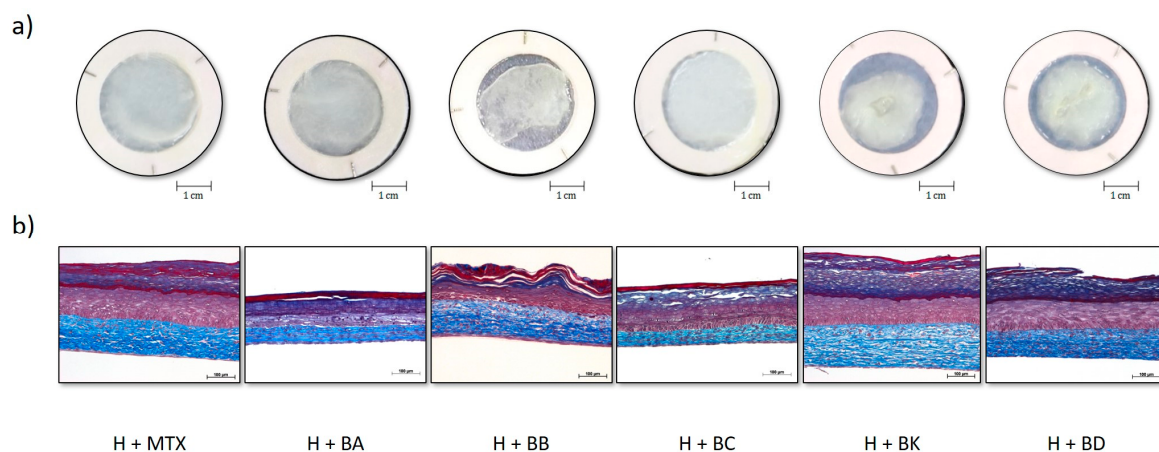

Supplement: Supplementary file 1 [file ijms-21-00256-s001.pdf]
